# Egg Usual Intake is Associated with Choline Adequacy in US Infants and Young Children

**DOI:** 10.1016/j.cdnut.2023.101958

**Published:** 2023-06-03

**Authors:** Yanni Papanikolaou, Victor L. Fulgoni

**Affiliations:** 1 Nutritional Strategies, Nutrition Research & Regulatory Affairs, Paris, ON, Canada; 2 Nutrition Impact, Nutrition Research, Battle Creek, MI, United States

**Keywords:** usual intake, eggs, choline, children, NHANES

## Abstract

Although most US children do not meet recommendations for choline intake, there are also no data available assessing usual egg intake in younger children and choline adequacy. Therefore, data from the National Health and Nutrition Examination Survey, 2001–2018 were analyzed to identify usual egg intake in infants (birth to 1 y; *N* = 4770) and young children (2–5 y; *N* = 6930) and to determine mean percentage of infants and children above the Adequate Intake (AI) for daily choline intake. The percent of infants above the AI when consuming the lowest usual egg intake level (<0.25 oz eq) was 33.4 ± 1.3. When comparing 0.25–0.5, 0.5–0.75, 0.75–1.0, and ≥1.0 oz eq to <0.25 oz eq of usual egg intake, the percent of infants above the AI for choline was 67.4 ± 1.6, 84.9 ± 2.1, 93.2 ± 1.5, and 98.1 ± 1.3, respectively (all *P* < 0.0001). The percent of children above the AI when consuming the lowest usual egg intake level (<0.25 oz eq) was 22.31. Comparing 0.25– 0.5, 0.5–0.75, 0.75–1.0, and ≥1.0 oz eq to <0.25 oz eq of usual egg intake demonstrated significant increases in the percent of toddlers above the AI for choline, such that 51.41%, 72.57%, and 84.94% and 92.57%, respectively, were above the recommended daily intake for choline (all *P* < 0.0001). Similar findings were seen when assessing infants and children of different socioeconomic status. Overall, the percent of infants and children above the AI was higher with each increasing level of usual egg intake. Given the association of higher choline intakes with egg consumption, increasing usual egg intake in infants and young children may help elevate the percentage meeting the established AI for choline intake and thus, improve choline adequacy in childhood.

## Introduction

The inclusion of eggs within American dietary pattern recommendations has been inconsistent over the last half of the century, with conventional scientific rationale around egg consumption routinely being associated with adverse health outcomes [1,2]. Limiting eggs within a healthy diet can be traced to the AHA guidance from the late 1960s to reduce egg consumption to <3 eggs per week to lower risk of CVD [3]. Recent Dietary Guidelines for Americans [4,5] as well as reviews [[6], [7], [8]] have re-examined and questioned previously established evidence around egg consumption and health and have proposed that previous dietary recommendations are no longer supported by current science and were predominantly based on misinterpreted and/or substandard scientific evidence. The Dietary Guidelines Advisory Committee (2020–2025) released food and nutrition recommendations for infants and toddlers that specifically recommended eggs as a first food in this age group, in addition to recommending eggs for preadolescents, adolescents, and pregnant and lactating women [9]. Eggs have also been identified as a cost-efficient source of dietary protein, choline, iron, zinc, riboflavin, and vitamin B12 while also contributing ∼140 kcal/100 g intake [10].

Recent analyses using data from the US NHANES demonstrate several nutrient intake benefits associated with egg consumption in the infant population. Egg consumption was significantly associated with higher intakes of protein, DHA, α-linolenic acid, lutein/zeaxanthin, total choline, and vitamin B12 in infant egg consumers compared with infant nonconsumers [11]. Similarly, NHANES data in children and adolescents showed positive associations with egg consumption and usual nutrient intakes, such that inclusion of eggs within dietary patterns was related to higher protein, total choline, potassium, selenium, phosphorus, DHA, α-linolenic acid, lutein and zeaxanthin, riboflavin, vitamin A, vitamin D, and vitamin E intakes, and lower total and added sugar intake, in comparison to children and adolescents avoiding eggs in dietary patterns [12].

Dietary guidance supports and encourages numerous protein-rich foods, including eggs, with the caveat that these foods be consumed with minimal added sugar, sodium, and solid fat [4,5]. The nutrient-dense attributes of eggs are well-documented, with one 50 g serving of egg providing numerous bioavailable nutrients [2,13], including being an important food source of choline, an underconsumed nutrient in the American population as indicated by the 2020 Dietary Guidelines Advisory Committee [14]. Recent work examining usual intakes of choline in US children of various age groups exhibited that both younger and older children do not meet authoritative guidance for dietary choline recommendations. Indeed, an NHANES modeling analysis showed that eggs play a substantial role in helping children meet choline recommendations, while in contrast, elimination of eggs from dietary patterns lowered dietary choline intake in all age groups, leading to fewer children above the established AI for choline [15].

With previous studies reporting higher intakes of choline in egg consumers, it was hypothesized that higher usual egg intakes would be associated with a higher percentage of US infants and younger children meeting choline intake recommendations, ultimately leading to an improvement in choline adequacy. Therefore, the objective of the current analysis was to determine association of usual intake of eggs with the percent of infants and younger children above the AI for choline intake. A secondary objective included assessing the primary objective in children of differing socioeconomic status.

## Methods

### Data source: US NHANES

Data for the current analysis were provided from the US NHANES data set, a cross-sectional survey designed to evaluate the nutritional and health in the free-living population. NHANES is a part of the NCHS, which is administered by the CDC [16]. All ethics protocols, including all proceedures mandated to obtain study consent from participants, have formerly been gathered and verified by the ethics committee of the CDC. Nine data sets were amalgamated for the study, including NHANES 2001–2002, 2003–2004, 2005–2006, 2007–2008, 2009–2010, 2011–2012, 2013–2014, 2015–2016, and 2017–2018 for children from birth to 5 y [17]. Egg consumption and choline intake data examined were from the relevant USDA Food and Nutrient Database for Dietary Studies database for NHANES [18]. The Food and Nutrient Database for Dietary Studies is used to determine nutrient intakes in What We Eat in America [19]. The What We Eat in America collection of data uses the Automated Multiple Pass Method, which has been recognized and documented as a valid, accurate, efficient, and evidence-based procedure for nationally representative dietary surveys [20]. As the present analysis considered dietary recalls in children aged <5 y, dietary recall information was gathered from a parent and/or guardian who was responsible for providing care. For the current research, the analysis focused on reliable and complete 24-h dietary recall data from day 1 (ie, in-person interview) and day 2 (ie, offsite follow-up interview via a telephone call) [21].

### Study participants and egg definitions

The analysis pooled data for all male and female children consuming eggs (birth to 1 y, *N* = 4770; 2 to 5 y, *N* = 6,930), in addition to conducting separate analyses for male and female participants dependent on socioeconomic status, as assessed by the poverty-income ratio (PIR). The PIR was grouped into 3 categories (<1.35, 1.35 ≤ PIR ≤ 1.85, and >1.85), reflecting the federally established poverty criteria; thus, a PIR of <1.35 equated to being in a lower income family, whereas higher PIR values represented the subject was from a higher income family.

The definition of egg intake focused on children consuming all egg food codes beginning with ‘311’ (ie, eggs, poached eggs, scrambled eggs, and omelets in the dietary recall), as well as the egg component in mixed dishes (ie, egg burritos, egg sandwiches, egg casseroles, egg breads, etc.) Eggs from subgroup 55 ‘Sweet Bakery Products’ (ie, cakes, cookies, pies, brownies, doughnuts, sweet rolls, and pastries) were excluded from the analysis as the egg contribution from these foods was insignificant.

### Egg usual intake assessment

Egg usual intake was determined using the methodology developed and validated by the NCI and has previously been described [22]. The study participants’ intake can significantly vary from one day to the next; thus, usual intakes are more appropriate and reflective of true intakes, enhancing accuracy when estimating nutrient adequacy. Assessments of nutrient adequacy using NHANES data are facilitated by statistical adjustment methods and have been previously documented. This method provides an estimate of the distribution of usual intakes from observed intakes, with the condition that >1 d of intake data are available to power the analysis [23]. Thus, both dietary recalls were used to estimate usual egg intake using the NCI model [22]. Subjects were separated into 5 usual egg intake (oz eq) groups:<0.25, 0.25–0.50, 0.50–0.75, 0.75–1.0, and ≥1.0 oz eq. The percent above the AI for dietary choline was determined using the cut-point method to provide a measure of individuals above the recommneded daily choline intake for each usual egg intake group [24].

### Statistical methodology

SAS 9.4 software was used to achieve all statistical analyses. Survey weights were incorporated to develop nationally representative estimates for study participants in addition to adjustments to reflect the complex sample design of NHANES [25]. Usual egg intake (oz eq) levels were identified (<0.25, 0.25–0.50, 0.50–0.75, 0.75–1.0, and ≥1.0 oz eq) and compared to the lowest usual egg intake level (ie, <0.25 oz eq). A *P* value of <0.01 was deemed to represent significance.

## Results

### Usual egg intake in infants and toddlers

Usual intake and usual egg distribution data are summarized in Table 1 by sex and by combined-sex results. Mean egg usual intake for infants (birth to 1 y) and children (2–5 y) were 0.23 ± 0.01 and 0.34 ± 0.01 oz eq. The median and 90th percentile of usual egg intake were 0.14 ± 0.01 and 0.55 ± 0.03, respectively, in those birth to 1 y and was 0.26 ± 0.01 and 0.69 ± 0.03, respectively in those 2–5 y.

**TABLE 1:** Usual intake and usual intake distribution of eggs (oz eq), NHANES 2001–2018, ages birth to 1 y and 2–5 y

Age, y	Sex	N	Usual intake		Usual intake percentiles													
			Mean	SE	P5	SE	P10	SE	P25	SE	P50	SE	P75	SE	P90	SE	P95	SE
0–1	All	4770	0.23	0.01	0.01	0.002	0.02	0.003	0.04	0.005	0.14	0.01	0.31	0.02	0.55	0.03	0.76	0.04
0–1	M	2461	0.24	0.01	0.01	0.002	0.02	0.003	0.05	0.01	0.14	0.01	0.32	0.02	0.58	0.03	0.78	0.04
0–1	F	2309	0.22	0.01	0.01	0.002	0.02	0.003	0.04	0.01	0.13	0.01	0.30	0.02	0.53	0.03	0.72	0.04
2–5	All	6930	0.34	0.01	0.06	0.01	0.09	0.01	0.15	0.01	0.26	0.01	0.44	0.01	0.69	0.03	0.89	0.04
2–5	M	3491	0.36	0.01	0.06	0.01	0.09	0.01	0.16	0.01	0.27	0.01	0.46	0.02	0.72	0.03	0.93	0.05
2–5	F	3439	0.33	0.01	0.06	0.01	0.08	0.01	0.14	0.01	0.25	0.01	0.42	0.02	0.66	0.03	0.85	0.05

Abbreviations: F, female; M, male; Mean, least square mean; oz eq, ounce equivalent; P, percentile; SE, standard error.

### Percent above the AI for choline intake: ages 0 to 1 y

Table 2 illustrates percent of infants and toddlers meeting total choline recommendations by egg consumption group. In sex-combined infants (birth to 1 y), the percent above the AI increased with each increasing level of usual egg intake. The percent of infants above the AI when consuming a usual egg intake of <0.25 oz eq was 33.4 ± 1.3. When comparing 0.25–0.5, 0.5–0.75, 0.75–1.0, and ≥1.0 oz eq to <0.25 oz eq of usual egg intake, the percent of infants above the AI for choline was 67.4 ± 1.6, 84.9 ± 2.1, 93.2 ± 1.5, and 98.1 ±1.3, respectively (all *P* <0.0001).

**TABLE 2:** Usual egg intake and mean percent above the AI for choline intake, NHANES 2001–2018, ages birth to 1 y and 2–5 y

Usual egg intake (oz eq)															
Sex	Age, y	<0.25 oz eq		0.25–0.5 oz eq		*P*	0.5–0.75 oz eq		*P*	0.75–1 oz eq		*P*	≥1 oz eq		*P*
		Mean	SE	Mean	SE		Mean	SE		Mean	SE		Mean	SE	
All	0–1	33.38	1.3	67.40	1.6	<0.0001	84.94	2.06	<0.0001	93.15	1.53	<0.0001	98.05	1.27	<0.0001
All	2–5	22.31	1.2	51.41	1.4	<0.0001	72.57	2.14	<0.0001	84.94	2.36	<0.0001	92.97	1.76	<0.0001
M	0–1	36.65	1.7	70.48	1.7	<0.0001	86.62	2.11	<0.0001	94.58	1.75	<0.0001	98.21	1.06	<0.0001
M	2–5	24.71	1.7	54.46	1.7	<0.0001	74.53	2.24	<0.0001	87.16	2.74	<0.0001	94.24	1.66	<0.0001
F	0–1	29.94	1.5	64.01	2.2	<0.0001	82.90	2.43	<0.0001	91.45	2.21	<0.0001	97.85	2.01	<0.0001
F	2–5	19.98	1.3	48.19	2.2	<0.0001	70.38	2.68	<0.0001	82.28	2.89	<0.0001	91.30	2.58	<0.0001

Abbreviations: F, female; M, male; Mean, mean percent above Adequate Intake (AI) for choline; oz eq, ounce equivalent; SE, standard error.

### Percent above the AI for choline intake: ages 2–5 y

In all children, the percent above the AI increased with each increasing level of usual egg intake. The percent of children above the AI when consuming a usual egg intake of <0.25 oz eq was 22.3. Comparing 0.25–0.5, 0.5–0.75, 0.75–1.0, and ≥1.0 oz eq to < 0.25 oz eq of usual egg intake demonstrated significant increases in the percent of toddlers above the AI for choline, such that 51.4 ± 1.4, 72.6 ± 2.1, 85.0 ± 2.4, and 93.0 ± 1.8, respectively, were above the recommended daily intake for choline (all *P* < 0.0001).

### Percent above the AI for choline intake: ages 0–1 y, PIR < 1.35

Table 3 shows the percent of infants and children meeting total choline recommendations by usual egg consumption group. All usual egg intake levels were compared with the lowest usual egg intake level (ie, <0.25 oz eq). Similar to data presented in Table 2, the percent of children above the AI was greater with each increasing level of usual egg intake. The percent of infants above the AI when consuming a usual egg intake of <0.25 oz eq was 35.1. When comparing 0.25–0.5, 0.5 –0.75, 0.75–1.0, and ≥1.0 oz eq to <0.25 oz eq of usual egg intake, the percent of infants above the AI for choline was 68.5 ± 1.8, 85.8 ± 1.9, 94.1 ±1.3, and 97.5 ± 1.1, respectively (all *P* < 0.0001).

**TABLE 3:** Usual egg intake and mean percent above the AI for choline intake, NHANES 2001–2018, ages birth to 1 y and 2–5 y, PIR < 1.35

Usual egg intake (oz eq)															
Sex	Age, y	<0.25 oz eq		0.25–0.5 oz eq		*P*	0.5–0.75 oz eq		*P*	0.75–1 oz eq		*P*	≥1 oz eq		*P*
		Mean	SE	Mean	SE		Mean	SE		Mean	SE		Mean	SE	
All	0–1	35.12	1.84	68.52	1.84	<0.0001	85.80	1.85	<0.0001	94.09	1.33	<0.0001	97.53	1.08	<0.0001
All	2–5	22.83	1.62	54.84	1.47	<0.0001	75.44	1.99	<0.0001	87.87	2.44	<0.0001	94.30	1.75	<0.0001
M	0–1	39.19	2.34	72.76	2.34	<0.0001	89.24	1.68	<0.0001	95.79	1.46	<0.0001	98.51	0.85	<0.0001
M	2–5	26.73	2.12	60.42	2.31	<0.0001	79.24	2.27	<0.0001	90.60	2.63	<0.0001	95.34	1.74	<0.0001
F	0–1	30.64	2.12	64.12	2.28	<0.0001	82.03	3.18	<0.0001	92.33	1.94	<0.0001	96.46	1.76	<0.0001
F	2–5	18.86	1.59	49.19	1.82	<0.0001	71.51	2.27	<0.0001	84.88	2.54	<0.0001	93.11	1.99	<0.0001

Abbreviations: F, female; M, male; Mean, mean percent above Adequate Intake (AI) for choline; oz eq, ounce equivalent; PIR, poverty-income ratio; SE, standard error.

### Percent above the AI for choline intake by age: ages 2–5 y, PIR < 1.35

Choline adequacy was elevated with increasing level of usual egg intake in children from households with PIR <1.35. The percent of children above the AI when consuming a usual egg intake of <0.25 oz eq was 22.8. Comparing 0.25–0.5, 0.5–0.75, 0.75–1.0, and ≥1.0 oz eq to <0.25 oz eq of usual egg intake demonstrated significant increases in the percent of toddlers above the AI for choline, such that 54.8 ± 1.5, 75.4 ± 2.0, 87.9 ± 2.4, and 94.3 ±1.8, respectively, were above the AI for choline intake (all *P* < 0.0001).

### Percent above the AI for choline intake: ages 0 to 1 y, 1.35 ≤ PIR ≤ 1.85

Table 4 shows the percent of infants and children above the AI for total choline by usual egg intake in children from households with 1.35 ≤ PIR ≤ 1.85. All usual egg intake levels were compared with the lowest usual egg intake level (ie, <0.25 oz eq). Similar to data presented in TABLE 2, TABLE 3, the percent of children above the AI was greater with each increasing level of usual egg intake, demonstrating an association between choline adequacy and usual egg intake. The percent of infants above the AI when consuming a usual egg intake of <0.25 oz eq was 34.6. When comparing 0.25–0.5, 0.5–0.75, 0.75–1.0, and ≥1.0 oz eq to < 0.25 oz eq of usual egg intake, the percent of infants above the AI for choline was 60.8 ± 6.7, 77.7 ± 8.1, 87.1 ± 10.7, and 91.8 ± 8.3, respectively (all *P* < 0.0001).

**TABLE 4:** Usual egg intake and mean percent above the AI for choline intake, NHANES 2001–2018, ages birth to 1 y and 2 to 5 y, 1.35 ≤ PIR ≤ 1.85.

Usual egg intake (oz eq)															
Sex	Age, y	<0.25 oz eq		0.25–0.5 oz eq		*P*	0.5–0.75 oz eq		*P*	0.75–1 oz eq		*P*	≥1 oz eq		*P*
		Mean	SE	Mean	SE		Mean	SE		Mean	SE		Mean	SE	
All	0–1	34.63	3.7	60.77	6.7	0.0001	77.69	8.1	<0.0001	87.07	10.7	<0.0001	91.76	8.3	<0.0001
All	2–5	24.84	5.3	47.53	3.8	0.0002	65.80	7.3	0.0002	74.48	8.6	<0.0001	85.25	10.1	<0.0001
M	0–1	38.05	5.0	64.96	7.4	0.0003	82.46	9.1	0.0000	90.29	10.4	<0.0001	90.13	8.6	<0.0001
M	2–5	27.01	6.0	51.46	5.1	0.0004	69.06	7.9	0.0001	78.47	9.1	<0.0001	89.84	12.4	0.0001
F	0–1	31.50	4.1	57.15	7.2	0.0001	74.20	8.5	<0.0001	84.78	11.6	<0.0001	93.33	11.0	<0.0001
F	2–5	22.18	5.3	43.24	4.0	0.0004	62.48	7.5	0.0003	70.54	9.1	<0.0001	80.91	11.4	0.0001

F, female; M, male; Mean, mean percent above Adequate Intake (AI) for choline; oz eq, ounce equivalent; PIR, poverty-income ratio; SE, standard error.

### Percent above the AI for choline intake by age and sex: ages 2 to 5 y, 1.35 ≤ PIR ≤ 1.85

Similar to the other age groups examined, choline adequacy was elevated with greater usual egg intake for those from households 1.35 ≤ PIR ≤ 1.85. The percent of children above the AI when consuming a usual egg intake of <0.25 oz eq was 24.8. Comparing 0.25–0.5, 0.5–0.75, 0.75–1.0, and ≥1.0 oz eq to <0.25 oz eq of usual egg intake demonstrated significant increases in the percent of toddlers above the AI for choline, such that 47.5 ± 3.8, 65.8 ± 7.3, 74.5 ± 8.6, and 85.3 ± 10.1, respectively, were above the AI for choline intake (all *P* < 0.0001).

### Percent above the AI for choline intake: ages 0 to 1 y, PIR > 1.85

When considering those from households with a PIR > 1.85, the percent of infants and toddlers above the AI for total choline by usual egg intake is summarized in Table 5. All usual egg intake levels were compared to the lowest usual egg intake level (ie, <0.25 oz eq). The percent of infants above the AI when consuming a usual egg intake of <0.25 oz eq was 32.8. When comparing 0.25–0.5, 0.5–0.75, 0.75–1.0, and ≥1.0 oz eq to <0.25 oz eq of usual egg intake, the percent of infants above the AI for choline was 66.8 ± 3.0, 84.8 ± 4.2, 93.6 ± 3.5, and 97.0 ± 2.5, respectively (all *P* ≤ 0.0001).

**TABLE 5:** Usual egg intake and mean percent above the AI for choline intake, NHANES 2001–2018, ages birth to 1 y and 2 to 5 y, PIR > 1.85.

Usual egg intake (oz eq)															
Sex	Age, y	<0.25 oz eq		0.25–0.5 oz eq		*P*	0.5–0.75 oz eq		*P*	0.75–1 oz eq		*P*	≥1 oz eq		*P*
		Mean	SE	Mean	SE		Mean	SE		Mean	SE		Mean	SE	
All	0–1	32.78	1.8	66.79	3.0	<0.0001	84.75	4.2	<0.0001	93.57	3.5	<0.0001	96.96	2.5	<0.0001
All	2–5	21.69	2.0	49.44	2.9	<0.0001	71.43	4.5	<0.0001	83.76	5.4	<0.0001	92.31	4.5	<0.0001
M	0–1	35.97	2.3	69.41	2.9	<0.0001	86.03	4.0	<0.0001	94.61	3.1	<0.0001	99.90	3.1	<0.0001
M	2–5	23.76	2.7	51.34	3.2	<0.0001	72.75	4.8	<0.0001	82.85	6.4	<0.0001	93.51	4.6	<0.0001
F	0–1	29.50	2.1	63.47	4.1	<0.0001	82.82	5.3	<0.0001	91.75	5.2	<0.0001	91.24	9.3	<0.0001
F	2–5	19.80	1.8	47.22	3.5	<0.0001	69.57	4.9	<0.0001	85.36	6.5	<0.0001	90.09	6.3	<0.0001

F, female; M, male; Mean, mean percent above Adequate Intake (AI) for choline; oz eq, ounce equivalent; PIR, poverty-income ratio; SE, standard error.

### Percent above the AI for choline intake: ages 2–5 y, PIR > 1.85

Similar to the other age groups and socioeconomic status examined, choline adequacy was elevated with greater usual egg intake in children from households with PIR >1.85. The percent of children above the AI when consuming a usual egg intake of <0.25 oz eq was 21.7. Comparing 0.25–0.5, 0.5–0.75, 0.75–1.0, and ≥1.0 oz eq to < 0.25 oz eq of usual egg intake demonstrated significant increases in the percent of toddlers above the AI for choline, such that 49.4 ± 2.9, 71.4 ± 4.5, 83.8 ± 5.4, and 92.3 ± 4.5, respectively, were above the AI for choline intake (all *P* ≤ 0.0002).

## Discussion

Certainly, eggs as part of a healthy dietary pattern can be an integral approach to help reduce shortfall gaps in choline intake and improve the likelihood of meeting recommendations in children. To our knowledge, this is the first study to examine egg usual intakes in younger American children with associations to choline adequacy. Greater usual egg intake was associated with a greater percentage of children above the AI for total choline intake. The lowest level of usual egg intake (<0.25 oz eq per day) in all infants showed that only ∼33% were above the AI for choline, whereas the mean percent of infants above the AI for choline increased to 98% when usual egg intake was ≥1 oz eq per day. When considering all children aged 2–5 y, regardless of socioeconomic status, increasing egg consumption was associated with a greater percentage above the AI for total choline. The lowest level of usual egg intake (<0.25 oz eq per day) showed that only ∼22% of children had intakes that were above the AI for choline, whereas the mean percent of children above the AI for choline increased to 93% when usual egg intake was ≥1 oz eq per day.

Choline has been recognized as an essential nutrient with significant scientific consensus to support a critical role in various physiologic mechanisms, including cell structure and signaling, neurotransmitter production, gene expression, lipid metabolism, and early brain and neural development [[26], [27], [28], [29]]. A recent randomized clinical trial demonstrated prenatal benefits with dietary choline supplementation. Indeed, choline supplementation in the second and third trimesters of pregnancy significantly improved DHA biomarkers, which play an essential role in fetal brain development [30]. Irrespective of the nutritional value of choline, most American children fall short of the established AI, with only very limited intake data available for infants and toddlers. Further, there is a lack of evidence evaluating choline usual intake within different developmental periods, with emphasis positioned on commencing studies to address research gaps in younger children. Recent work examining choline intake in healthy Canadian toddlers at ages 1 and 2 y showed the majority of subjects examined were not meeting the recommended AI of 200 mg per day, with the highest food contributors of choline being animal-derived (ie, dairy, meats, and eggs) [31]. Estimated total choline intakes per day were similar in previous studies when assessing US toddlers (229 mg/d) [32] and European toddlers (151–210 mg/d) [33].

Previous work from our group showed that modeling the removal of eggs from the diet of infants and younger children substantially lowered dietary choline intake, which translated into a reduced percentage of children above the AI for choline. In contrast, further analyses revealed the addition of 1 egg per week to the current US dietary pattern resulted in nearly 10% more infants above the AI for choline. Similarly, modeling the consumption of 1 egg per day within the current typical dietary pattern showed that nearly 100% of US infants would exceed the AI for choline. In older children, modeling the addition of 1 egg per day to the current dietary pattern resulted in ∼95% of participants surpassing the dietary recommendations for choline [15].

Observational studies similar to the present analysis using NHANES data have limitations inherent within epidemiologic studies and have been reported in earlier research [[34], [35], [36]]. Nevertheless, the use of NHANES to assess nutrition and public health outcomes offers considerable strengths. Indeed, the use of a large, cross-sectional survey like NHANES contributes a distinct advantage in allowing researchers access to a large cross-sectional database that combines refined personal and individual assessments with biochemical, clinical, and anthropometric examinations for various age groups, ethnicities, and socioeconomic status. Shortcomings of the modeling approach using NHANES include recollection bias, which includes the over- or underreporting of foods and beverages consumed. Nonetheless, NHANES has employed protocols that are considered the gold standard for collecting dietary intake data that have been previously documented [37,38]. Although the current study used the AI for choline as established by the Food and Nutrition Board of the National Academies of Sciences, Engineering, and Medicine, it is necessary to state that current AI for choline in children was extrapolated from 1 study in adult males with the primary objective of assessing total choline intake and the prevention of hepatic changes [24]. Thus, it is reasonable to speculate that the inferred choline AI for young children may need to be further investigated due to the high level of growth and development occurring in early childhood. Finally, the current analysis did not verify if subjects younger than 6 mo consumed solid foods.

In conclusion, to our knowledge, our analysis provides the first evidence of associations between usual egg intake in American children and percentage of the population with choline intakes above the AI. The current data further support that eggs, as part of a healthy dietary pattern, can be advantageous in helping reduce shortfall gaps in choline intake in children. Greater usual egg intake was associated with a greater percentage of children above the AI for total choline intake in all ages and socioeconomic levels examined. Public health strategies, school nutrition programs, and authoritative dietary guidance should further consider the nutritional significance of eggs within healthy dietary patterns in children to help achieve choline recommendations.

## Funding

Supported by the Egg Nutrition Center. The Egg Nutrition Center did not contribute to any part of the intellectual design, data analysis, concept development, or manuscript drafting of the research study. Furthermore, the Egg Nutrition Center had no role in determining publication of the present findings.

## Author disclosures

YP serves as President of Nutritional Strategies and is responsible for partnering with the food, beverage, and vitamin/mineral supplement industry on nutrition- and regulatory affairs-related initiatives. Both VLF and YP routinely work in partnership on nutrition projects encompassing data from the US National Health and Nutrition Examination Survey. VLF serves as SVP of Nutrition Impact and works with food, beverage, and vitamin/mineral companies to advance nutrition research scientific knowledge.

## Data availability

Data described in the manuscript, code book, and analytic code will be made available upon request.
